# The Influence of TiO_2_–Lignin Hybrid Fillers in Low-Density Polyethylene Composites on Photocatalytic Performance and UV-Barrier Properties

**DOI:** 10.3390/polym16040474

**Published:** 2024-02-08

**Authors:** Patryk Jędrzejczak, Michał Cegłowski, Karol Bula, Łukasz Klapiszewski

**Affiliations:** 1 Faculty of Chemical Technology, Institute of Chemical Technology and Engineering, Poznan University of Technology, PL-60965 Poznan, Poland; patryk.jedrzejczak@doctorate.put.poznan.pl; 2 Faculty of Civil and Transport Engineering, Institute of Building Engineering, Poznan University of Technology, PL-60965 Poznan, Poland; 3 Faculty of Chemistry, Adam Mickiewicz University, PL-61614 Poznan, Poland; michal.ceglowski@amu.edu.pl; 4 Faculty of Mechanical Engineering, Institute of Material Technology, Poznan University of Technology, PL-60965 Poznan, Poland; karol.bula@put.poznan.pl

**Keywords:** kraft lignin, titanium dioxide, hybrid material, polyethylene, polymer composites

## Abstract

The main objective of this study was to discover new packaging materials that could integrate one of the most expected properties, such as UV protection, with a self-cleaning ability defined as photocatalytic performance. Accordingly, new hybrid additives were used to transform LDPE films into materials with complex performance properties. In this study, titanium dioxide–lignin (TL) hybrid systems with a weight ratio of inorganic to organic precursors of 5-1, 1-1, and 1-5 were prepared using a mechanical method. The obtained materials and pristine components were characterized using measurement techniques and research methods, such as Fourier-transform infrared spectroscopy (FTIR), thermal stability analysis (TGA/DTG), measurement of the electrokinetic potential as a function of pH, scanning electron microscopy (SEM), and particle size distribution measurement. It was found that hydrogen bonds were formed between the organic and inorganic components, based on which the obtained systems were classified as class I hybrid materials. In the next step, inorganic–organic hybrid systems and pristine components were used as fillers for a low-density polyethylene (LDPE) composite, 5 and 10% by weight, in order to determine their impact on parameters such as tensile elongation at break. Polymer composites containing titanium dioxide in their matrix were then subjected to a test of photocatalytic properties, based on which it was found that all materials with TiO_2_ in their structure exhibit photocatalytic properties, whereby the best results were obtained for samples containing the TiO_2_–lignin hybrid system (1-1). The mechanical tests showed that the thin sheet films had a strong anisotropy due to chill-roll extrusion, ranging from 1.98 to 3.32. UV–Vis spectroscopy revealed four times higher light absorption for composites in which lignin was present than for pure LDPE, in the 250–450 nm range. On the other hand, the temperature at 5% and 30% weight loss revealed by TGA testing increased the highest performance for LDPE/TiO_2_ materials (by 20.4 °C and 8.7 °C, respectively).

## 1. Introduction

In recent years, significant effort has been made to use lignocellulosic biomass as a raw material to produce energy, chemicals, and materials in a sustainable and environmentally friendly way [1,2]. The use of lignocellulosic biomass in the plastics industry has particularly high potential. By 2050, global plastic production is estimated to increase to 1.1 billion tons [3], with approximately 40% of plastics used for storing and packaging finished products. This is due to a number of properties that plastics have, e.g., low cost and resistance to chemical agents [4,5]. The most popular are polyolefins, including low-density polyethylene (LDPE) and high-density polyethylene (HDPE), as well as isotactic polypropylene (PP) [5].

Nowadays, materials with improved properties are becoming increasingly common. To obtain them, nanoparticles of various substances are introduced into the polymer matrix, and the resulting polymer composites may have, among other features, better mechanical properties, lower gas permeability, and increased microbiological resistance [5]. In some cases, introducing fillers into the polymer matrix also has economic reasons [6]. A variety of products are used to produce polymer composites, including biopolymers, such as lignin [1,7,8,9,10,11,12] and cellulose [13,14,15,16,17,18,19]; inorganic substances, e.g., titanium dioxide [20,21,22,23]; and hybrid materials, e.g., MgO/lignin [24] or TiO_2_/lignin [25].

Among organic fillers for polymer composites, lignin and its derivatives have recently attracted great interest. This is due, among other things, to the fact that it appears in huge quantities in the biosphere; estimated at approximately 350 billion tons [1]. Moreover, the advantage of using the mentioned biopolymer is the fact that lignin is an inexpensive, natural, and biodegradable material, which is a waste product in the paper industry and in biorefineries [7]. However, polyolefins and lignin in their original natural form, due to the highly hydrophilic nature of the biopolymer, are immiscible; therefore, additional modifications are required to change the properties of lignin and improve the dispersion of its particles in the polymer matrix [7,24].

In the 21st century, thanks to the intensive development of nanotechnology, new perspectives have appeared in the development of materials based on polymeric materials. Various nanometric, inorganic compounds, such as titanium dioxide [21,26,27] or zinc oxide [27], can be used as fillers for nanocomposites. For example, in the work of Youssef et al., nanometric particles of titanium dioxide were used as a filler for LDPE, which resulted in an increase in the crystallinity of the polymer matrix, improvement in mechanical properties and thermal stability, as well as obtaining antimicrobial activities [21]. Also, in Kavuncuoglu’s work, TiO_2_ was used as a filler for LDPE due to its low toxicity, low cost, and photocatalytic and antimicrobial properties. As part of the conducted research, the use of nanometric particles of titanium dioxide was found to contribute to obtaining lower values of elongation at break compared to the reference sample, which is related to the fact that nanometric oxides create weak points during tensile tests and limit the mobility of polymer chains. This work also noted that when increasing the amount of nanometric oxide introduced into the polymer matrix, the tendency to form agglomerates increases, which may contribute to the deterioration of the mechanical parameters of the nanocomposite [27].

An innovative approach is using hybrid systems as fillers for polymer composites. Creating a hybrid system that includes an organic and inorganic component allows one to obtain a material with improved properties in relation to the components used [25]. Organic–inorganic hybrid systems can be divided into two classes, depending on the strength of the bonds between the components. In the case of weak interactions, i.e., van der Waals forces, hydrogen bonds, and electrostatic interactions, the material is classified as class I hybrid material. In turn, the presence of strong covalent bonds between the organic and inorganic components indicates that a class II hybrid material has been obtained [28]. Using organic–inorganic hybrid materials as fillers for polymer composites enables the creation of products with better parameters. For example, in the work of Zielińska et al., a polypropylene nanocomposite containing a TiO_2_/nanocellulose hybrid system in its matrix was obtained. The obtained product was characterized by very high tensile strength and higher Young’s modulus values and showed high nucleation activity and thermal resistance [29].

Recently, the authors made several attempts to test hybrid fillers (inorganic particles combined with kraft lignin) as modifiers of the properties of packaging polymers. Significant improvements in barrier properties, i.e., oxygen permeability and water vapor permeability, were achieved, with percentage decreases of 18 and 56, respectively, especially for composites where a MgO/lignin hybrid system (1:5 wt./wt.) was incorporated into LDPE [24]. In subsequent tests, the seal strength/durability was tested to estimate the performance of the sealing joint [30]. For example, the study revealed that the seal open force of LDPE reinforced with MgO/lignin (1:5 wt./wt.) increased by more than 30% compared to LDPE [31]. The positive role of the combined hybrid filler has been proven in a practical evaluation regarding the thermoformability of composite materials, which was assessed by comparing wall thickness distributions of thermoformed shapes. Again, the best material performance was obtained for composites with a hybrid filler and, importantly, with the highest lignin loading in the two-phase filler [32]. The above examples have proven that lignin, in combination with selected metal oxides, plays a very positive role in determining the properties of packaging materials, which can be used in a wide range of packaging applications.

The main purpose of this attempt is that, for the first time, we plan to test the activity of titanium dioxide particles combined with lignin and embedded in LDPE film for photocatalytic performance. This combination of inorganic/organic additives has never been tested before in such an application. Therefore, new recipes were determined for hybrid fillers in which the combination of TiO_2_ and kraft lignin was properly designed and obtained using a mechanical method. In addition, such new systems will be tested for their potential photocatalytic properties, including instances of UV-barrier properties. Also of interest is the discovery of the effect of the hybrid filler system on the mechanical properties and anisotropy of films produced by the uniaxial drawing procedure.

## 2. Materials and Methods

### 2.1. Materials

Kraft lignin purchased from Merck KGaA, Darmstadt, Germany (average Mw: ~10,000 g/mol, CAS number: 8068-05-1) and titanium dioxide in the anatase crystallographic form (CAS number: 1317-70-0), supplied by Sigma-Aldrich (Steinheim am Albuch, Germany), were used to produce polymer composites in this publication.

Low-density polyethylene (LDPE) Malen E grade, described as FGNX 23-D006, from Basell Orlen, Płock, Poland, was used as a polymer matrix. This grade revealed a mass melt flow rate (MFR) of 0.8 g/10 min at 190 °C, and it is appropriate for the manufacture of highly transparent very fine films. The films with a thickness of 40 microns have good mechanical properties and are distinguished by their high transparency and gloss. 

As a compatibilizing agent, a polar component polyethylene–graft–maleic anhydride copolymer was used, supplied by Merck & Co., Inc. (Rahway, NJ, USA), with 0.5% maleic anhydride content.

### 2.2. Preparation of TiO_2_–Lignin Hybrid Systems

In order to combine titanium dioxide and kraft lignin, mechanical grinding of pristine components with their simultaneous mixing was performed using (i) RM100 mortar grinder (Retsch GmbH, Haan, Germany) for 1 h and then (ii) Pulverisette 6 Classic Line ball mill (Fritsch GmbH, Idar-Oberstein, Germany) for 1 h. The combined application enables the creation of a final material characterized by adequate homogeneity. After milling, the powder was sieved through a sieve with a diameter of 80 µm. TiO_2_–lignin hybrid systems were prepared with the following weight ratio of inorganic to organic parts: 5-1, 1-1, and 1-5.

### 2.3. Characteristics of TiO_2_–Lignin Hybrid Systems

Thermal stability analysis of the obtained products was performed using a Jupiter STA 449F3 analyzer (Netzsch GmbH, Selb, Germany). All samples were weighed at approximately 10 mg, placed in an Al_2_O_3_ crucible, and heated in a nitrogen atmosphere at a rate of 10 °C/min from 30 to 800 °C.

The morphology and microstructure of the obtained samples were analyzed using photomicrographs obtained with a Tescan VEGA3 scanning electron microscope (SEM) (Tescan Orsay Holding a.s., Brno, Czech Republic). Additionally, particle size distributions and the polydispersity index were calculated using a Zetasizer Nano ZS instrument (Malvern Instruments Ltd., Malvern, UK), which operates based on the non-invasive backscattering (NIBS) technique and is capable of measuring particle sizes in the range of 0.6–6000 nm.

The electrokinetic stability of the materials was investigated using a Zetasizer Nano ZS instrument equipped with an MPT-2 autotitrator (Malvern Instruments Ltd., Malvern, UK). The measurements use a combination of electrophoresis and laser measurement of particle mobility based on the Doppler effect. This is the electrophoretic light scattering (ELS) method, based on measurement of the rate of migration of particles in a liquid under the influence of an electric field, which is called electrophoretic mobility. The zeta potential is not measured directly, but is calculated from experimentally obtained electrophoretic mobility values using the Henry equation. The samples were prepared by dispersing 10 mg of the appropriate product in 25 mL of a 0.001 M NaCl solution, and then titrated with a 0.2 M HCl solution or a 0.2 M NaOH solution until the desired pH was obtained, which was monitored using a glass electrode. Measurements were made in a pH range from 2 to 10. The mean measurement error of the zeta potential was ±2 mV, and the measurement error of the pH value was ±0.1.

Characteristic functional groups on the surface of the analyzed samples were identified using Fourier-transform infrared spectroscopy (FTIR) working in transmission mode. The measurement used a Vertex 70 spectrometer containing the LMCT detector (Bruker Optics GmbH & Co. KG, Ettlingen, Germany). The FTIR spectra were obtained in the transmission mode between 4000 and 450 cm^−1^ and at a resolution of 1 cm^−1^. The TiO_2_–lignin hybrid systems and pristine components were measured in the classical transmission mode while using potassium bromide (KBr) as the background.

### 2.4. Preparation of Polyethylene-Based Composites

The processing of compositions containing low-density polyethylene, compatibilizer PE-g-MAH, and selected additives based on TiO_2_, kraft lignin, and hybrid formulation started with the mixing of components in a powder state in a mixing chamber with a ribbon-like mixer. The content of single additives and hybrid formulas in each polyethylene-based sample series is listed in Table 1, as are sample abbreviations. Prepared batches of mixed components were dried in an air-circulating oven at 95 °C for 4 h. Melt processing of polymeric compounds was realized in a twin-screw extruder, in co-rotating mode, equipped with a 16 mm screw diameter, and operated at a barrel temperature of 165–200 °C and a screw rotation speed of 150 rpm (Zamak 16/40 EHD, Skawina, Poland). Some detailed information about screw configuration was described in our previous paper [33]. The produced strands were subsequently cooled in a conveying belt with cooling fans without using a water bath that prevented secondary moisture absorption by the lignin phase. The extrudates were then re-grounded to the form of granulate with laboratory grinder. The pellets of LDPE and granulated composite formulations were then cast-extruded using a single-screw extruder, Metalchem 28/30 (Metalchem Sp. z o.o., Gliwice, Poland) (with a screw diameter of 28 mm and an L/D ratio of 30) and a semi-laboratory chill-roll device (Remi-Plast, Czerwonak, Poland). The temperature at the plasticizing unit was set between 180 and 210 °C from feed to die, with a main screw rotation speed of 45 rpm. The shaping of thin films was realized with a 1 mm thick and 250 mm long slit die. The thin films of polyethylene and its composites were processed at constant chill-roll speed set to 2.5 m/min, while chill-roll temperature was set to 40 °C and stabilized by an external cooling thermocontroller device. Prior to mechanical testing, the films were conditioned at 23 °C and 50% RH for 48 h to obtain a stable structure.

### 2.5. Characteristics of Polyethylene-Based Composites

#### 2.5.1. Mechanical Properties

A uniaxial tensile test (tensile strength, elongation at break, and modulus) was performed using a universal testing machine equipped with a 10 kN load cell (Zwick Roell Group 010 Z, Ulm, Germany). The films were cut into rectangular specimens (strips of 10 mm width and 200 mm length) in the transverse direction (TD) and along the extrusion direction (MD—machine extrusion) and tested according to [34] at a crosshead speed of 200 mm/min. For each series, at least 9 samples were subjected to mechanical tests. Of the results obtained, the minimum and maximum results were rejected.

#### 2.5.2. Thermal Analysis

The thermogravimetric analysis according to [35] was performed using a Netzsch TG 209 F1 Libra (Netzsch GmbH, Selb, Germany). Samples of approximately 10 mg were placed in an Al_2_O_3_ crucible and heated at a rate of 10 °C/min from 30 to 900 °C in a nitrogen atmosphere at a flow rate of 25 cm^3^/min.

#### 2.5.3. UV–Vis Spectrophotometry Analysis

The optical properties of the composite films were determined by measuring light absorption at 200–900 nm, at the optical resolution of 4 nm, using a UV–Vis spectrophotometer (UV line 9400, Schott Instruments, Mainz, Germany).

#### 2.5.4. Photocatalytic Properties

In order to assess the photocatalytic properties, fragments of 4 cm × 4 cm were cut out of the produced films that were then attached to polypropylene plates. In the next step, the prepared systems were placed in quartz beakers, and 50 mL of phenol solution was poured at a concentration of 10 mg/L (phenol was a model organic pollutant in this experiment). A magnetic dipole was then placed in the beaker to ensure continuous mixing of the system, and the beaker was sealed by covering it with a quartz watch glass. The prepared system was placed on a magnetic stirrer and stirred in the dark for 24 h to check the percentage of contamination absorbed onto the composite surface. After ensuring that the absorption did not exceed 5%, the photocatalytic properties were tested. This involved placing the test system under an LED lamp emitting ultraviolet radiation with a wavelength of 395 nm. The lamp was supplied by Bridgelux (Fremont, CA, USA) and was made using chip-on-board technology with a power of 50 W. Additionally, it was equipped with an active cooling system consisting of an aluminum radiator and a fan. The presence of these device elements is intended to remove the heat generated during the process and ensure stable temperature conditions during the photocatalytic properties test. The lamp was placed 20 cm above the sample, and the UV radiation intensity was 500 ± 10 mW/cm^2^. After placing the beaker with the test sample on a stirrer under an LED lamp and placing it in the darkroom, the test began by turning on the radiation source. The test was carried out for 6 and 24 h, after which a sample of 3 mL was collected. The sample was filtered using syringe filters (Macherey-Nagel, Duren, Germany).

The phenol concentration was determined utilizing the Vanquish HPLC system (Thermo Fisher Scientific, Waltham, MA, USA), featuring a C18 chromatography column (Accurore C18; particle size 2.6 µm) and a UV–Vis detector equipped with a DAD photodiode array. The experimental conditions were as follows: column temperature of 45 °C, flow rate of 0.3 cm^3^/min, and mobile phase composition of 50% methanol and 50% water (*v*/*v*).

The kinetics of phenol photodegradation were assessed using a pseudo-first-order kinetic model. According to this model, it was assumed that the rate of degradation of the model organic pollutant is directly proportional to the surface coverage (*θ*) by phenol, expressed as follows (1):

(1) $$r=\frac{dC}{dt}=k\theta =\frac{{kKC}_{0}}{1+KC_{0}+K_{S}C_{S}}$$

where: k is the rate constant of the reaction, K and $K_{S}$ are the adsorption coefficients of phenol and water, $C_{0}$ is the initial concentration of phenol, and $C_{S}$ is the concentration of water. Since the concentration of water remains nearly constant and is significantly higher than the concentration of phenol, the equation can be rearranged in the following form (2):

(2) $$\ln\frac{C_{t}}{C_{0}}=-k_{1}t$$

In Equation (2), $C_{t}$ represents the concentration of phenol after t time of irradiation, and $k_{1}$ is the first-order reaction rate constant.

## 3. Results and Discussion

### 3.1. Characteristics of TiO_2_–Lignin Hybrid Systems and Pristine Components

#### 3.1.1. Thermal Stability Assessment

**Figure 1:**
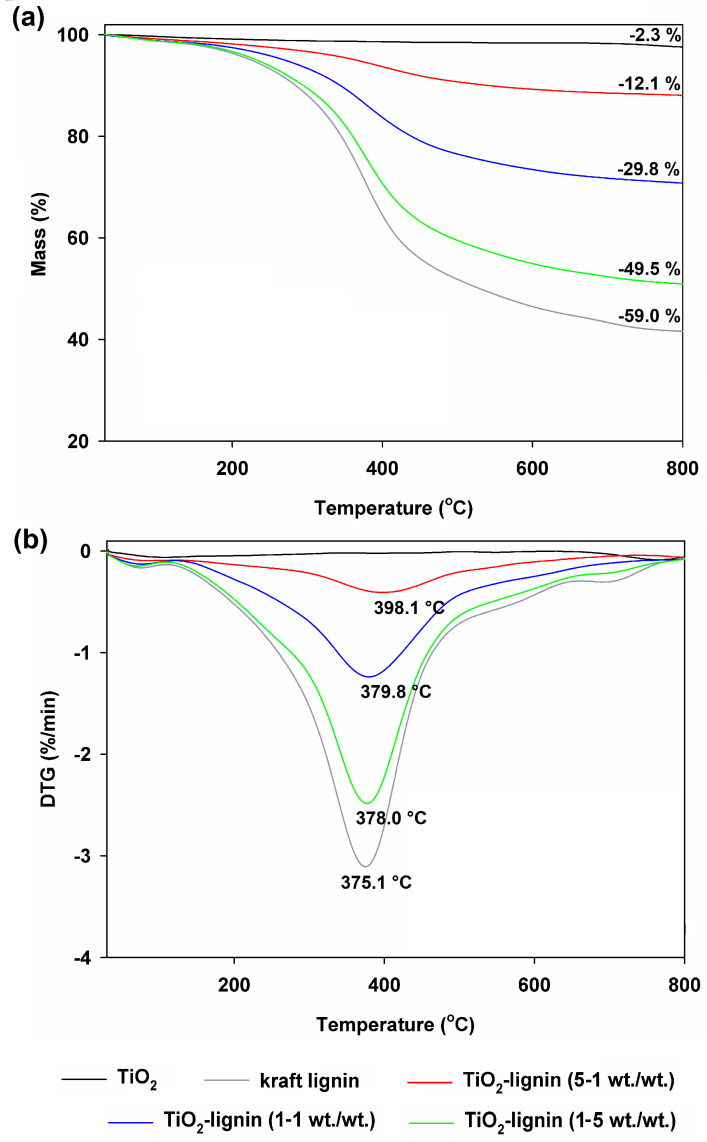
Thermogravimetric (TGA) curves (**a**) and derivative thermogravimetric (DTG) curves (**b**) of TiO_2_–lignin hybrids and pristine components (titanium dioxide and kraft lignin).

A certain limitation when using kraft lignin as a filler for polymer composites is its thermal stability. For this reason, it was important to check the thermal stability of the kraft lignin and titanium dioxide used and the resulting hybrid systems. The data obtained through thermal analysis are presented as thermograms and differential thermogravimetric curves in Figure 1.

Comparing the thermogravimetric curves, it can be observed that the most thermally stable sample was the pristine inorganic precursor, while kraft lignin has relatively limited thermal stability. The mass loss of these materials in the temperature range of 30–800 °C was 2.3% and 59.0%, respectively. The higher the share of inorganic precursor in the sample, the greater the thermal stability of TiO_2_–lignin hybrid systems. As a result of the analysis performed in the case of hybrid materials, the following mass losses were observed: 12.1%, 29.8%, and 49.5% obtained for the TL(5-1), TL(1-1), and TL(1-5) products, respectively. As can be seen, as a result of combining titanium dioxide with kraft lignin, hybrid systems were created that showed greater thermal stability than the pristine organic precursor, thus increasing the potential of using the biopolymer as a filler for polymer composites. Analyzing the differential thermogravimetric curves, it can be seen that the main mass loss occurs at temperatures ranging from 350 to 420 °C. In the case of kraft lignin, the greatest mass loss occurs at a temperature of 375.1 °C, while for hybrid materials, this value shifts toward higher temperatures as the share of the inorganic part increases. With regard to products marked TL(1-5), TL(1-1), and TL(5-1), the largest mass losses were found for 378.0 °C, 379.8 °C, and 398.1 °C, respectively. This is related to the increase in thermal stability as a result of combining kraft lignin with titanium dioxide using hydrogen bonds, which indirectly confirms the effectiveness of the production of TiO_2_–lignin hybrid systems.

#### 3.1.2. Characteristics of Dispersion and Morphological Properties

**Figure 2:**
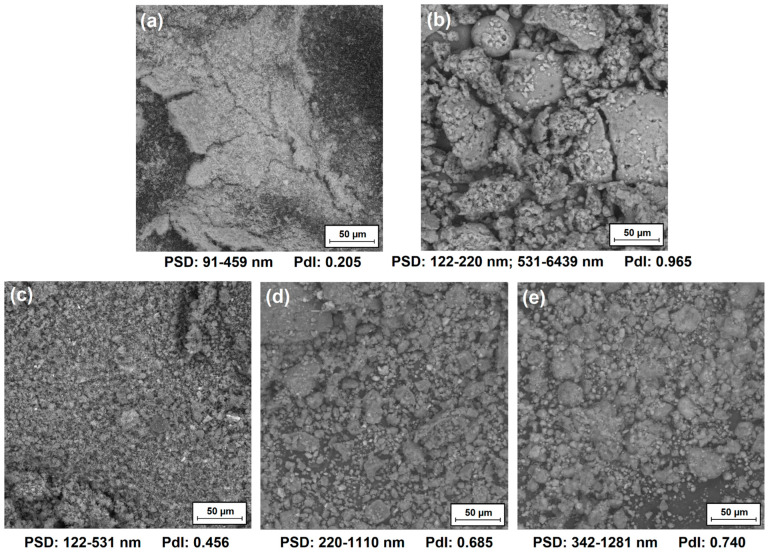
SEM images, particle size distributions (PSD), and polydispersity indexes (PdI) of titanium dioxide (**a**), lignin (**b**), and TiO_2_–lignin hybrids with a weight ratio of components equal to 5-1 (**c**), 1-1 (**d**), and 1-5 (**e**).

As part of this work, three TiO_2_–lignin (TL) hybrid systems were obtained with a weight ratio of inorganic to organic precursor of 5-1, 1-1, or 1-5. It is important to determine the dispersion and morphological properties of these materials and pristine components. Figure 2 shows micrographs obtained using scanning electron microscopy of all of the analyzed samples, i.e., TiO_2_ (T) and lignin (L) components and the TL(5-1), TL(1-1), and TL(1-5) hybrid systems. Additionally, this figure shows the ranges of the particle size distributions of these materials and polydispersity indices, which were determined using the Zetasizer Nano ZS apparatus. 

Analyzing the obtained SEM micrographs of the components, both materials tend to aggregate and agglomerate, but the lignin particles are much larger than the relatively small particles of titanium dioxide. In the case of hybrid systems, the tendency of these materials to create larger aggregate and agglomerate structures was also observed. Smaller particles were observed in the case of hybrids with a higher content of titanium dioxide. In the case of titanium dioxide, particle size is an important parameter because TiO_2_ exhibits particle size-dependent properties, e.g., photocatalytic activity. Moreover, the presence of large particle clusters—as in the case of a pristine organic precursor, i.e., kraft lignin—in the polymer matrix may contribute to the weakening of such a composite.

The conclusions drawn by analyzing SEM micrographs of pristine components and hybrid systems were also confirmed by analyzing the particle size distributions of these products. It was observed that titanium dioxide in the anatase form has particles with the smallest sizes, ranging from 91 to 459 nm, while the particles of the second precursor reach sizes even above 6 µm. In the case of hybrid systems, it was observed that as the share of the organic component increases, the particle size ranges shift toward larger values, and for subsequent products, TL(5-1), TL(1-1), and TL(1-5) are, respectively, 122–531 nm, 220–1110 nm, and 342–1281 nm.

Another important parameter in the case of the analyzed materials is the polydispersity index. Analyzing the polydispersity indexes of the TiO_2_ and TL(5-1) samples, which are 0.205 and 0.456, respectively, it can be concluded that the particles of these materials are much more uniform in size than hybrid systems with a higher content of kraft lignin.

#### 3.1.3. Electrokinetic Stability Assessment

The combination of kraft lignin with titanium dioxide in a hybrid system has a significant impact on the electrokinetic stability of the resulting systems. For this reason, in this study, the electrokinetic potential was determined as a function of pH, and the obtained data are summarized in Table 2. Analyzing the electrokinetic potential values, both the TL(5-1), TL(1-1), and TL(1-5) hybrid systems and the pristine components, i.e., the TiO_2_ and L values of the electrokinetic potential, decrease with decreasing acidity.

The measurement of the electrokinetic potential can determine whether a given material forms electrokinetically stable dispersions at a given pH. A dispersion is considered electrokinetically stable when the electrokinetic potential is greater than +20 mV or less than −20 mV. All analyzed samples form electrokinetically stable dispersions at neutral and alkaline pH, while at pH = 2, only TiO_2_ has the ability to create such dispersions. Combining titanium dioxide with kraft lignin in hybrid systems enables the creation of materials with lower electrokinetic potentials and thus can create more electrokinetically stable dispersions. Among the analyzed products, the most electrokinetically stable material is the TiO_2_–lignin system with a weight ratio of inorganic to organic precursor of 1-5. The electrokinetic potential of this sample is −30.1 mV at pH = 4 and −40.8 mV at pH = 10. At alkaline pH, the other two hybrid systems also achieve lower electrokinetic potentials of −36.6 mV and −37.1 mV for TL(5-1) and TL(1-1), respectively, compared to the organic precursor (−30.8 mV) and titanium dioxide (−30.1 mV).

#### 3.1.4. Fourier-Transform Infrared Spectroscopy

**Figure 3:**
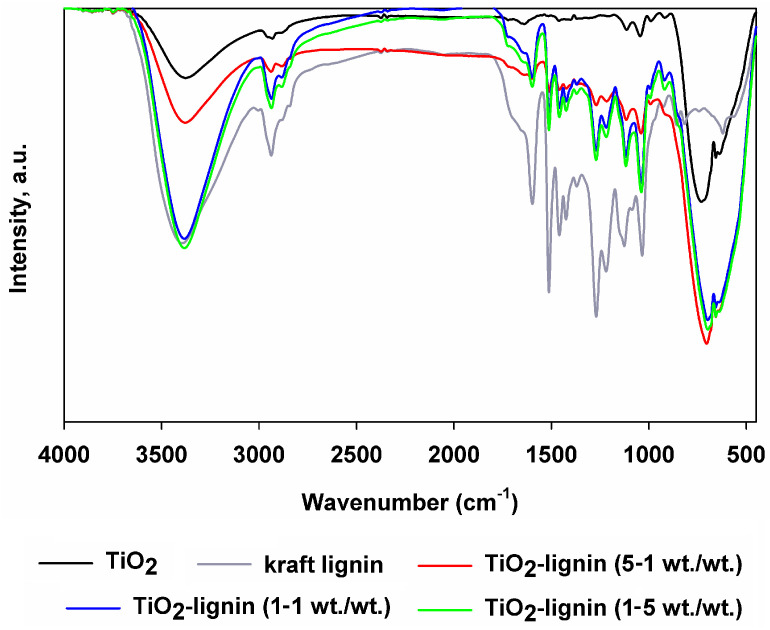
Fourier-transform infrared spectra of TiO_2_–lignin hybrids and pristine components (titanium dioxide and kraft lignin).

To determine the functional groups present in the structure of the components used and the hybrid systems obtained with their participation, Fourier-transform infrared spectroscopy was used (see Figure 3). In the case of the inorganic component, a band at the wavenumber of 3400 cm^−1^ was observed, originating from the stretching vibrations of O-H groups, most likely related to physically bound water molecules on the surface of the photocatalyst [36]. However, the band with a maximum in the range of 785–500 cm^−1^ is associated with stretching vibrations of the Ti-O and Ti-O-Ti groups [37,38]. The remaining visible bands in the FTIR spectrum of titanium dioxide most likely come from impurities remaining after the TiO_2_ synthesis process. The next analyzed infrared spectrum was that obtained for kraft lignin. Typical bands of functional groups present in the structure of this biopolymer were observed in earlier works [37,39,40,41,42]. Namely, the band at the wavenumber 3423 cm^−1^ originates from the stretching vibrations of hydroxyl groups. Subsequently, a band of medium intensity was observed with a maximum at 2937 cm^−1^ and a band of low intensity at 2883 cm^−1^, characteristic of the stretching vibrations of C-H groups occurring in methyl and methylene groups, respectively. The low-intensity band at the wavenumber 2843 cm^−1^ is most likely related to the vibrations of the -O-CH_3_ groups of methoxy groups. At 1680 cm^−1^, a band resulting from the vibrations of C=O groups is visible. The next sharp bands with maxima at wavenumbers 1595 cm^−1^, 1510 cm^−1^, 1458 cm^−1^, and 1423 cm^−1^ come from the aromatic vibrations of C-C and C=C groups and are related to the presence of aromatic rings in the structure of kraft lignin. Due to the stretching vibrations of the C-O groups, a band with a maximum of 1267 cm^−1^ is visible. The next two bands at the wavenumbers 1367 cm^−1^ and 1217 cm^−1^ are characteristic of the bending vibrations of the Ar-OH groups of phenolic groups and the stretching vibrations of the C-O groups of carbons present in aliphatic chains and aromatic rings.

Furthermore, the FTIR spectra of hybrid systems contain bands originating from the same groups as in the components used. The differences that can be observed appear in the intensity of these bands, which is related to the different weight ratios of the organic to inorganic component in the structure of the resulting hybrid material. The shifts in the maximum of the bands are related to the occurrence of hydrogen bonds between the components. These bonds were created when titanium dioxide was combined with kraft lignin, allowing the obtained materials to be classified as class I inorganic–organic hybrid systems [25]. Thanks to the analysis of FTIR spectra and the presence of hydrogen bonds between the organic and inorganic components, it can be concluded that the proposed mechanical method of producing these materials was effective. 

The presented statements resulting from the analysis of FTIR spectra coincide with the literature data regarding the synthesis of inorganic–organic systems such as SiO_2_/lignin [30,33] and MgO/lignin [31,32].

### 3.2. Characteristics of Polyethylene-Based Composites

#### 3.2.1. Mechanical Properties

**Figure 4:**
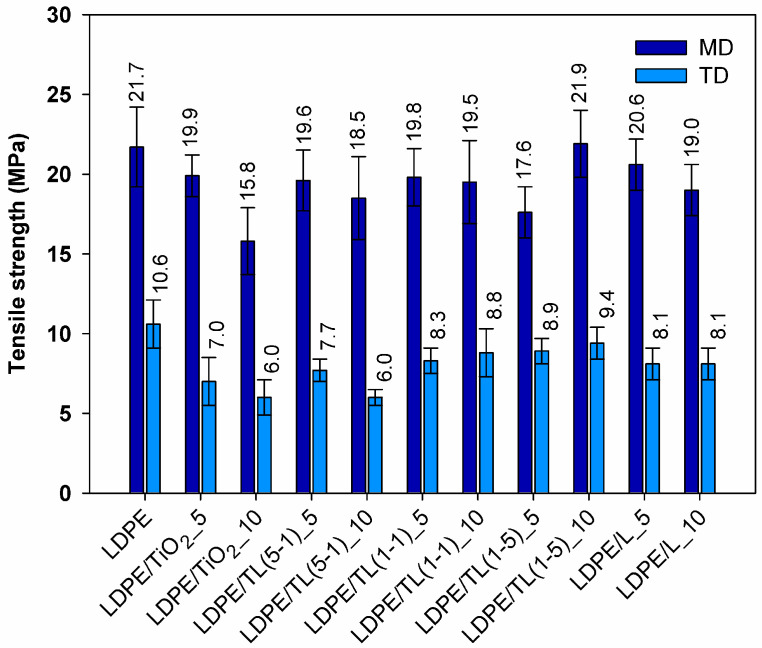
Tensile strength for samples cut out from the sheets in the regimes of machine direction (MD) and transverse direction (TD).

**Figure 5:**
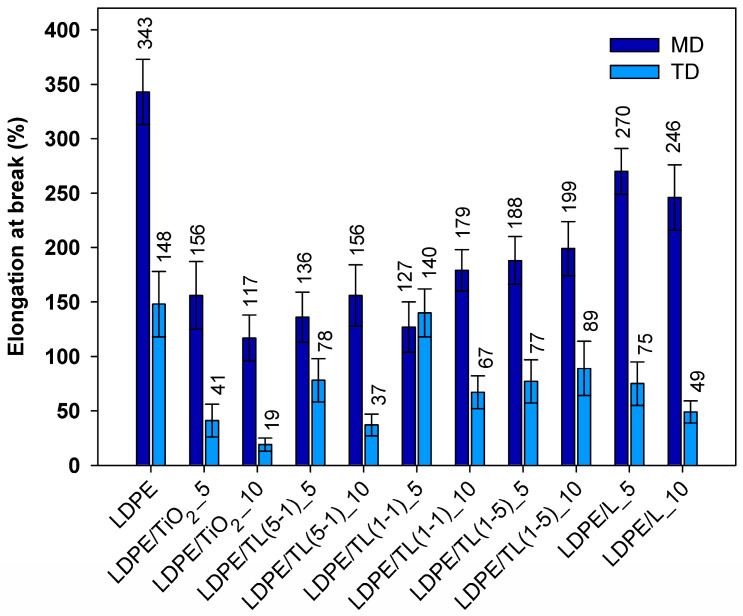
Elongation at break for samples cut out from the sheets in the regimes of machine direction (MD) and transverse direction (TD).

**Figure 6:**
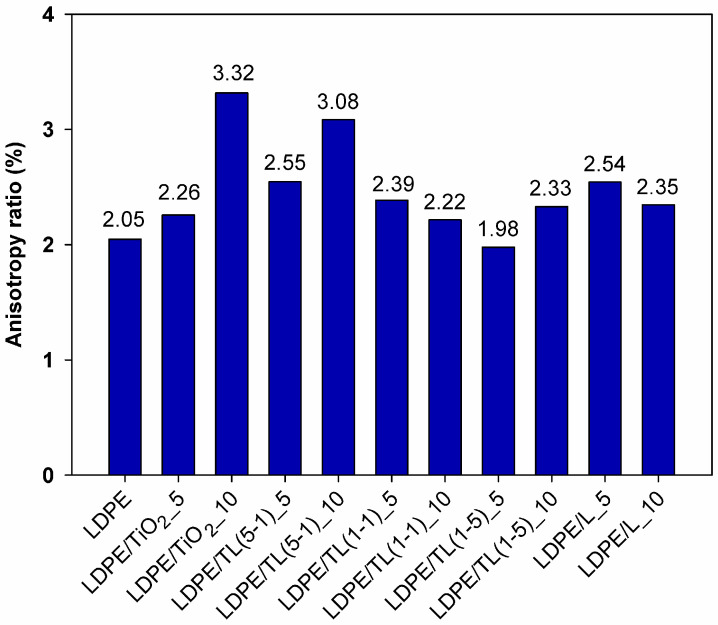
Anisotropy ratio in the case of tensile strength.

The tensile properties of LDPE and LDPE/TiO_2_–lignin composites obtained from thin strips with the thickness in the range of 80–150 µm and elongated at a constant rate are reported in Figure 4 and Figure 5. For packaging-like materials, important properties include the ultimate strength, elongation at break, and the anisotropy ratio, which is related to mechanical properties in machine and transverse directions. Comparing the ultimate tensile strength for samples cut in the machine direction, the highest values were noticed for LDPE and for the composite with 10% by wt. of a hybrid structure with a 1–5 wt./wt. ratio. Among tested composites, only the sample with a 10% load of TiO_2_ revealed a noticeable decrease in tensile strength, which could be explained as an agglomerate factor or rigid filler loading factor. It can also be observed that the values of tensile strength of the samples cut out from the thin sheets in the transverse direction (TD) are over two times lower compared to those taken along the extrusion direction (MD). This feature comes from the preferred orientation during film extrusion and, in this case, is also promoted by the strong uniaxial drawing of the polymer macromolecules during film formation using the chill-roll winding method. Therefore, the anisotropy ratio (AR) of the tested films is a very important parameter that provides additional information about the products that are produced at the cast line; the results are summarized in Figure 6. 

For two kinds of materials, the AR factor is close to the 2% value; these samples include neat LDPE and its composite with the 5% hybrid filler with a 1–5 wt./wt. ratio. The highest anisotropy ratio was read out for composites with a 10% load of TiO_2_ and a 10% load of hybrid filler with a 5-1 wt./wt. ratio. Adding fillers, in which titanium dioxide is the predominant additive, reduces the ability of polyethylene to orient, which leads to a reduction in the strength of TD samples compared to those of PE. It can be assumed that, from a practical point of view, the aforementioned composites are, for example, less suitable for thermoforming processes and should provide uneven tensile deformation. As a result, thermoformed parts are likely to exhibit high mechanical anisotropy. The highest value of elongation at break is manifested by the LDPE sample (Figure 5). Out of all of the tested composites, the best elongation at break was found in two samples where only lignin was incorporated. This behavior may be related to the action of lignin as a sliding agent. Low-molecular-weight lignin chines can penetrate between entangled polymer molecules and promote their mobility. Short lignin molecules dispersed in the polyethylene structure can increase the mobility of polymer chains, and the elongation in this case is not aggravated by lignin inclusions. Such a positive effect on lignin action was also noticed in polypropylene/lignin composites [33].

#### 3.2.2. Thermal Analysis

The TGA procedure was used to compare the thermal properties of pure LDPE film and films cast-extruded from composite materials containing pristine titanium dioxide and lignin particles, as well as hybrid filler additives. The important parameters resulting from the thermal degradation process were determined using TGA curves and the derivative of weight loss (DTG) curves. Adequate relevant temperature characteristics are aggregated in Table 3, including T_onset_ (the temperature at which the sample weight loss becomes more apparent), T_max_ (the temperature at which the degradation rate is fastest), and T_s_ (statistic heat-resistant index temperature). To determine the T_s_ value, the temperature at 5% weight loss—T_d5_ and 30% weight loss—T_d30_ should be read from the TGA curves. The T_s_ value was calculated based on Equation (3) [43]:

T_s_ = 0.49 [T_d5_ + 0.6(T_d30_ − T_d5_)] (3)

As shown in Table 3, for all composites noted, the T_max_ values shifted to higher temperatures with respect to LDPE. The shift in T_max_ is particularly evident as the lignin content of the composite increases, as well as for the hybrid fillers. After adding 5% and 10% TiO_2_ by wt., composite films showed a substantial increase in T_d5_ and T_d30_ values (with 20.4 °C and 8.7 °C, respectively, for LDPE/TiO_2__10 formulation). This feature is probably due to the very high thermal stability of titanium dioxide (see Figure 1). As a result, these composites showed the best static heat resistance index. Noteworthily, the thermally stable inorganic additive also has a positive effect in all tested hybrid systems.

#### 3.2.3. UV–Vis Spectrophotometry

**Figure 7:**
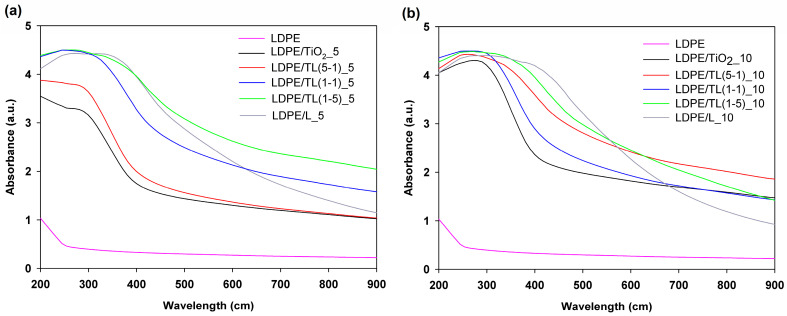
UV–Vis spectra of LDPE-based composite films: (**a**) at a 5% by wt. concentration of the filler and (**b**) at a 10% by wt. filler presence.

Ultraviolet and visible spectrometers were used to verify the ability of the new composites to absorb light and reveal their protective properties. The visible and ultraviolet light absorption curves of the composites in the wavelength range of 200–900 nm are shown in Figure 7. In this spectrum, it is clear that LDPE has marginal absorption in the wavelength range greater than 220 nm, which was also noted by [44]. By including pristine TiO_2_ particles, the presence of a broad absorption peak between 300 and 350 nm is evidenced. For LDPE composites with hybridized TL (5-1 wt./wt.), the absorption curve has the same character described for the LDPE/TiO_2_ material. With regard to the other composite films tested, only a broad band from 200 to 500 nm is observed in the absorbance range. This is probably due to the very strong absorption of lignin and their aromatic groups and conjugated films carbonyl groups [45,46]. UV traces for LDPE/lignin composites show two shoulders and two absorption bands, which are clearly visible at a concentration of 5% by weight and 10% by weight. The first is attributed to the presence of the C=O chromophore in the lignin chain (around 250 nm), while the second is due to the presence of phenolic groups (in the 400–450 nm range) [47]. The light absorption of the LDPE composite films gradually decreases above 500 nm. In general, by increasing the lignin content in the hybrid filler system, as well as in the pristine lignin additive, we can assume that such composites reveal a great possibility for ultraviolet light absorption.

#### 3.2.4. Photocatalytic Properties

**Figure 8:**
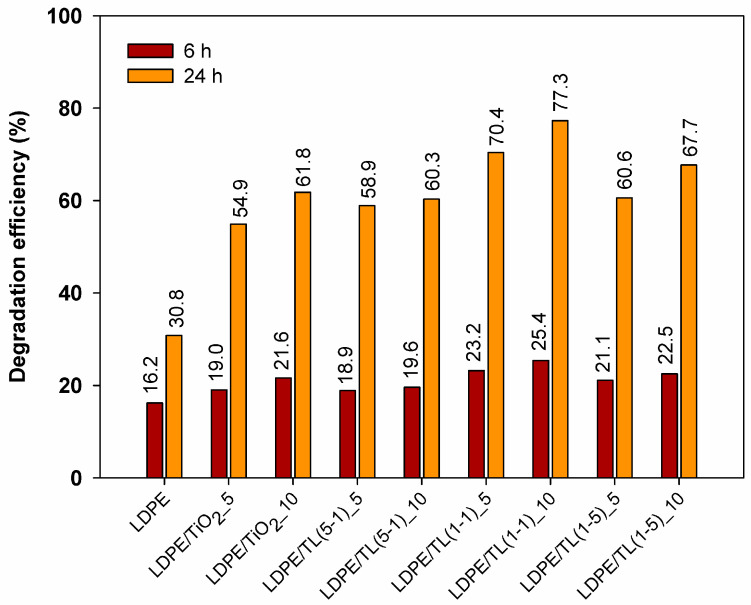
Degradation efficiency of pure LDPE and its composite films.

Titanium dioxide is known for its very good photocatalytic properties, so it was used to test the photocatalytic activity of the selected LDPE composites. The obtained data are presented in Figure 8. The calculated kinetics parameters of photocatalytic phenol degradation are presented in Table 4. Due to the introduction of TiO_2_ and TiO_2_-based hybrid materials, the rate constant *k*_1_ of the reaction increases compared to the reference material LDPE. The highest rate constants were observed for the LDPE/TL(1-1)_5 and LDPE/TL(1-1)_10 samples, amounting to 8.39·10^−4^ and 1.02·10^−3^, respectively. In the case of 6 h of exposure, differences in the degradation of the model organic pollutant between samples were observed, amounting to over nine percentage points. The obtained degradation efficiencies of the model organic pollutant range from 16.2% to 25.4%. After the mentioned time, the lowest degradation efficiency was found for the LDPE reference sample, and the highest was for the sample containing 10% by weight of the TL(1-1) hybrid system in its matrix. However, after 24 h of irradiation, the differences between samples in terms of degradation efficiency become even greater and amount to 46.5 percentage points between the most active sample (LDPE/TL(1-1)_10) (77.3%) and the reference sample (30.8%). It can be observed that the introduction of titanium dioxide and TiO_2_–lignin hybrid systems into the LDPE polymer matrix contributes to the creation of materials showing greater photocatalytic activity in the degradation reaction of the model organic pollutant compared to the reference sample. The highest degradation efficiency of the model organic pollutant, i.e., phenol, was obtained for the LDPE/TL(1:1)_5 and LDPE/TL(1:1)_10 samples, which were 70.4% and 77.3%, respectively. Comparing samples containing 5% and 10% by weight of a given filler, it should be expected that the photocatalytic activity will significantly improve due to increasing the amount of photocatalyst in the polymer matrix. However, based on the results obtained, it was found that an increase in the share of material with photocatalytic properties in the composite structure contributes to an increase in the degradation efficiency of the model organic pollutant, but only in the range of 1.4 to 7.1 percentage points. This is most likely because, with too high a filler content in the polymer matrix, the tendency of these materials to aggregate and agglomerate increases. Given the larger amount of TiO_2_ in the polymer matrix, the radiation cannot reach all exposed photocatalyst particles due to the shielding effect.

Based on the results of the photocatalytic activity of polymer composites, the introduction of twice as large amounts of TiO_2_ or hybrid systems TL(5-1), TL(1-1), and TL(1-5) is only justified to a limited extent. Polymer composites containing hybrid systems in their structure show better photocatalytic properties than the LDPE/TiO_2__5 and LDPE/TiO_2__10 samples because, by combining the photocatalyst with kraft lignin in the hybrid, a material capable of better dispersion in the polymer matrix is obtained. Moreover, for correct photocatalysis, it is important that as much of the photocatalyst as possible is located on the surface of the composite and not “immersed” in its structure, where the access of excitation radiation and substances capable of reacting with the free electrons and holes produced is limited. The presence of lignin in the hybrid material most likely promotes both of these phenomena.

The literature contains works on LDPE polymer composites with photocatalytic properties and containing titanium dioxide particles in their structure. In the work by Han et al., thin, flexible, and durable low-density polyethylene films were obtained, containing 30% by weight titanium dioxide in their structure. In the next step, the resulting composites were irradiated with UV radiation to be used to deactivate a number of different viruses, including SARS-CoV-2. In order to obtain the active material in this work, the LDPE film containing TiO_2_ had to be activated by prior exposure to ultraviolet radiation for 144 h. As a result of pre-conditioning, titanium dioxide particles present on the surface or at a short distance from it photocatalytically oxidize the thin polyethylene layer covering them, resulting in the creation of a photocatalytically active surface. It was then found that for each tested virus, the concentration of the active substance dropped to a very low level after 2 h of exposure [48]. However, in the work by Osorio-Vargas et al., polyethylene composites exhibiting photocatalytic properties in visible light were produced. This was achieved by synthesizing graphitic carbon nitride-TiO_2_ nanorods from titanium dioxide. Moreover, in this work, the photocatalyst was applied to the surface of the LDPE film using a method based on photocatalytic immobilization. This allowed the production of an LDPE film exhibiting photocatalytic properties under the influence of visible light [49]. Moreover, Alvear-Daza et al. decided to deposit the photocatalyst on the surface of a foil made of low-density polyethylene using photocatalytic immobilization. The material responsible for the photocatalytic properties were nanometric particles of titanium dioxide P25. By exposing the obtained foils to ultraviolet radiation, it was found that they exhibit good photocatalytic properties as well as superhydrophilicity [50].

## 4. Conclusions

In this work, an attempt was made to obtain inorganic–organic TiO_2_–lignin hybrid materials with a component weight ratio of 5-1, 1-1, or 1-5. Based on the analysis of infrared spectra, the effectiveness of obtaining these materials was confirmed, and hydrogen bonds were successfully formed between titanium dioxide and lignin. Therefore, the obtained hybrid systems were classified as class I hybrid materials. Moreover, the obtained hybrid systems are characterized by greater thermal stability than pristine kraft lignin. All hybrid materials and pristine precursors create electrokinetically stable dispersions in neutral and alkaline environments. Additionally, all tested systems show relatively good homogeneity.

As part of the research, pristine components and hybrid systems were introduced into low-density polyethylene at 5 and 10% by weight. It is worth commenting that composites with hybrid systems, especially lignin, revealed slight deterioration of tensile strength. This may prove their thermal stability during processing and was confirmed in the TGA analysis by revealing a statistical heat resistance index. This index increased for all composites with hybrid fillers compared to LDPE. In addition, there was a plasticizing effect of lignin demonstrated through a good elongation capacity compared to other hybrid filler materials. Under uniaxial drawing, the TiO_2_ composites showed high mechanical anisotropy, while the LDPE/TL(1-5)_5 composite achieved the lowest anisotropy value. UV spectroscopy once again proved the very positive role of the conjugated lignin structure in light-barrier resistance, thus showing that samples containing titanium dioxide or TiO_2_–lignin hybrid systems in the matrix exhibit photocatalytic activity. The highest efficiency in the degradation of the model organic pollutant, i.e., phenol, amounting to 77.3 and 70.4%, was obtained for LDPE/TL(1-1)_10 and LDPE/TL(1-1)_5 samples, respectively.

To summarize, using TiO_2_–lignin hybrid systems as a filler for an LDPE composite, materials with photocatalytic properties were obtained. Such systems can be used in engineering and medical applications, e.g., to obtain products that retain their original properties, such as their aesthetic values, for longer.

## Figures and Tables

**Table 1:** Composition of the produced polymer composites.

Sample	Weight Percentage			Filler Used
	LDPE	Filler	PE-g-MAH	
LDPE	98	0	2	-
LDPE/TiO_2__5	93	5		T (TiO_2_)
LDPE/TiO_2__10	88	10		
LDPE/TL(5-1)_5	93	5		TL(5-1)
LDPE/TL(5-1)_10	88	10		
LDPE/TL(1-1)_5	93	5		TL(1-1)
LDPE/TL(1-1)_10	88	10		
LDPE/TL(1-5)_5	93	5		TL(1-5)
LDPE/TL(1-5)_10	88	10		
LDPE/L_5	93	5		L (lignin)
LDPE/L_10	88	10		

**Table 2:** Zeta potential vs. pH for TiO_2_–lignin hybrid materials and pristine components.

Sample	pH				
	2	4	6	8	10
	Zeta Potential (mV)				
TiO_2_	25.2	−8.9	−18.4	−28.5	−30.1
kraft lignin	−6.5	−20.1	−23.3	−26.1	−30.8
TiO_2_-lignin (5-1 wt./wt.)	18.3	−12.3	−26.1	−30.5	−36.6
TiO_2_-lignin (1-1 wt./wt.)	−1.3	−28.5	−32.3	−36.2	−37.1
TiO_2_-lignin (1-5 wt./wt.)	−10.3	−30.1	−36.1	−38.2	−40.8

**Table 3:** TGA and DTG data of pure LDPE and its composites films.

Sample	T_onset_	T_max_	T_d5_	T_d30_	T_s_
	°C				
LDPE	466.9	476.2	429.8	466.0	221.2
LDPE/TiO_2__5	460.3	477.2	443.0	472.9	225.9
LDPE/TiO_2__10	469.8	479.3	450.2	474.7	227.8
LDPE/TL(5-1)_5	461.3	476.9	441.7	472.2	225.4
LDPETL(5-1)_10	471.6	482.8	440.6	471.7	225.0
LDPE/TL(1-1)_5	470.4	478.1	439.5	470.2	224.4
LDPE/TL(1-1)_10	459.2	483.1	437.0	469.5	223.7
LDPE/TL(1-5)_5	475.4	484.4	437.0	469.5	223.7
LDPE/TL(1-5)_10	466.0	481.5	433.1	470.2	223.1
LDPE/L_5	459.9	486.1	431.0	469.2	222.4
LDPE/L_10	467.1	485.1	423.0	468.9	220.7

**Table 4:** The calculated kinetic parameters for the photocatalytic degradation of model organic pollution (phenol).

Sample	*k*_1_ (1/min)	R^2^
LDPE	2.69 × 10^−4^	0.962
LDPE/TiO_2__5	5.55 × 10^−4^	0.999
LDPE/TiO_2__10	6.69 × 10^−4^	0.999
LDPE/TL(5-1)_5	6.15 × 10^−4^	0.999
LDPE/TL(5-1)_10	6.39 × 10^−4^	0.999
LDPE/TL(1-1)_5	8.39 × 10^−4^	0.999
LDPE/TL(1-1)_10	1.02 × 10^−3^	0.997
LDPE/TL(1-5)_5	6.47 × 10^−4^	0.999
LDPE/TL(1-5)_10	7.80 × 10^−4^	0.999

## Data Availability

Data are contained within the article.
